# Comparative proteomics of bark and xylem provides insights into age-dependent corticular photosynthesis in *Eucalyptus grandis*

**DOI:** 10.3389/fpls.2026.1701400

**Published:** 2026-02-04

**Authors:** Felipe Alexsander Rodrigues da Silva, Daniele Cristine de Lima, Mônica T. Veneziano Labate, Ilara Gabriela Frasson Budzinski, Thais Regiani Cataldi, Carlos Alberto Labate

**Affiliations:** Max Feffer Laboratory of Plant Genetics, Department of Genetics, College of Agriculture “Luiz de Queiroz”, University of São Paulo, Piracicaba, Brazil

**Keywords:** carbon metabolism, corticular photosynthesis, eucalyptus, fermentation, glycolysis, proteomics

## Abstract

**Introduction:**

Eucalyptus species are globally important for forestry due to rapid growth, adaptability, high biomass production, and contribution to carbon sequestration by storing atmospheric CO_2_ as biomass. However, the metabolic mechanisms sustaining growth under hypoxic conditions within woody vascular tissues remain unclear. Here, we investigate whether corticular photosynthesis helps sustain stem energy metabolism across two developmental stages in vascular tissues of *Eucalyptus grandis*.

**Methods:**

We analyzed bark and xylem from 4- and 12-year-old clonal *Eucalyptus grandis* plants. Chloroplast abundance in bark was quantified by fluorescence microscopy, and both tissues were profiled by shotgun proteomics.

**Results:**

Chloroplasts were more abundant in younger bark and were not detected in xylem. A total of 3,113 non-redundant proteins were identified, and enrichment analysis indicated a consistent hypoxic response across tissues and ages, alongside age-specific metabolic processes. Proteoform abundance patterns implicated glycolysis, the tricarboxylic acid cycle, and fermentation pathways. Alcohol dehydrogenase and aldehyde dehydrogenase proteoforms showed differential abundance in xylem and younger bark, consistent with greater emphasis on fermentative metabolism in hypoxia-prone vascular tissues. Younger bark also exhibited higher abundance of Calvin–Benson cycle proteins, together with higher chloroplast numbers than older bark and xylem, indicating higher potential for local carbon fixation and oxygen availability in juvenile stems.

**Discussion:**

These findings underscore adaptive metabolic strategies of eucalyptus stems, refine current models of corticular photosynthesis and stem energy metabolism in fast-growing trees, and provide a molecular framework for future physiological studies in eucalyptus and other woody species.

## 1 Introduction

Eucalyptus species are among the most widely cultivated trees worldwide, playing an important role in global forestry and wood production. They are known for their rapid growth, particularly in the first 2–4 years after planting, reaching 12–15 m of height. The *Eucalyptus* genus is extensively used in the production of pulp, paper, and bioenergy worldwide (Gonçalves et al., 2013; Li et al., 2023). Brazil has approximately 7.8 million hectares dedicated to eucalyptus and is one of the largest eucalyptus producers in the world, contributing significantly to the country’s economy and environmental sustainability efforts (Indústria Brasileira de Árvores (IBÁ), 2024). Moreover, eucalyptus plantations also contribute to carbon sequestration, playing an essential role in mitigating climate change by capturing atmospheric CO_2_ and storing it as biomass (Morales et al., 2023).

The biological mechanisms underlying energy metabolism in woody plants remain underexplored. Vascular tissues, such as phloem and xylem, comprehend the main bioproduct of interest in woody plants, thus exhibiting unique metabolic adaptations to meet the energetic demands for growth and maintenance (Dominguez and Niittylä, 2022), particularly under conditions of limited oxygen availability (León et al., 2020). Understanding how eucalyptus tissues sustain energy production in the early years of development is critical for advancing our knowledge of tree growth, carbon allocation, and resilience to environmental stress (Kong et al., 2021; Eloy et al., 2017).

For a better understanding of biomass accumulation and carbon sequestration, studies of carbon metabolism are fundamental since it is the main mechanism for plant growth and development. Glycolysis, the tricarboxylic acid (TCA) cycle, and fermentation pathways are central to energy generation and the production of metabolic intermediates essential for cellular processes (Chaudhry and Varacallo, 2023; Arnold et al., 2022; Fredlund et al., 2006; Bernacki et al., 2023). In the xylem (or cambial zone), where oxygen availability can be limited due to dense cell structures and root respiration, hypoxia may trigger different plant responses (De Roo et al., 2020). In addition, such metabolic adaptations are crucial for sustaining energy to the plant, especially rapid growth trees such eucalyptus (Hinchee et al., 2009). Corticular photosynthesis further supports the energetic demands of woody tissues by recycling respiratory CO_2_ and generating oxygen (Wittmann and Pfanz, 2014). By producing oxygen locally, corticular photosynthesis could alleviate hypoxic stress in vascular tissues, promoting efficient mitochondrial respiration and energy production (Kocurek et al., 2020).

Therefore, proteomics can be a powerful tool to unveil the role of the main proteins involved in primary metabolism. The knowledge regarding different regulatory proteins in vascular tissues across contrasting developmental stages may be applicable for monitoring metabolic responses of woody plants for better understanding xylogenesis and assisting plant breeding programs. Here, we employed a proteomic approach to investigate the metabolic differences between the bark and xylem of *Eucalyptus grandis* stems at two developmental stages: 4 and 12 years old. Our results revealed distinct protein abundance patterns associated with corticular photosynthesis, glycolysis, fermentation, and the TCA cycle, highlighting age/tissue-specific metabolic adaptations. Notably, xylem and younger bark tissues revealed more abundant alcohol dehydrogenase (ADH) and aldehyde dehydrogenase proteoforms, suggesting that the fermentative pathway works as a supporting mechanism to sustain adenosine triphosphate (ATP) production under hypoxic conditions. These findings provide novel insights into the metabolic plasticity of woody tissues and underscore the importance of corticular photosynthesis and alternative energy pathways in supporting eucalyptus growth and homeostasis. In *Eucalyptus*, proteome-level evidence connecting corticular chloroplast abundance with age-dependent shifts in primary carbon and energy metabolism across bark and xylem is scarce. By combining chloroplast quantification with comparative proteomics at two developmental stages, we provide a molecular framework and a set of candidate metabolic signatures that can be used to formulate and test explicit hypotheses on oxygen-related metabolism during xylogenesis.

## 2 Material and methods

### 2.1 Plant material and experimental conditions

Trees of a commercial plantation of *E. grandis* clone SP1318, kindly provided by Suzano Pulp and Paper, at two different developmental stages (4 and 12 years old) were analyzed during the summer of 2022. The clone was planted at a spacing of 3 × 2 m under standard silvicultural management practices. In total, there were eight trees at different developmental stages (four 4-year-old and four 12-year-old trees). Samples were collected from two different experimental stations: Fazenda de São Roque—Tatuí, São Paulo, Brazil (23°23′24.56″ S, 47°46′50.74″ W) and Fazenda Estrelas—Alambari, São Paulo, Brazil (23°32′28″ S, 47°50′44″ W) for 4- and 12-year-old trees, respectively. Both sites are located in the same region of the state of São Paulo and share similar climatic conditions. Importantly, all trees were harvested on the same day within a 2-h interval, so that both age classes experienced comparable environmental and light conditions at the time of sampling. For all trees, samples were consistently collected from the same height (breast height) and from the same relative position on the trunk, ensuring a comparable orientation with respect to sun exposure. In addition, stem panels extending from bark to xylem were collected from the same position in the trunk for histological analyses of chloroplast distribution.

Each tree was considered a biological replicate and samples of bark and xylem were collected from both age groups. Four biological replicates were considered for each tissue and developmental stage, totaling 16 biological samples. Harvesting the vascular tissues followed the methodology described by Celedon et al. (2007). Briefly, the bark of each tree was removed at chest height, and the exposed stem tissue and the inner surface of the bark were then scraped with a razor blade. All samples were immediately frozen in liquid nitrogen and stored at −80°C until further analysis.

### 2.2 Chloroplast distribution analysis

To estimate chloroplast distribution, transverse sections from bark to xylem were obtained in the field and subsequently processed into 80-µm-thick sections using a Leica SM 2000 R sliding microtome. For such analysis, three technical replicates were prepared for each biological replicate, and the histological sections were mounted on slides and analyzed using fluorescence microscopy at 10× and 20× magnifications. Chloroplast abundance and distribution were determined based on autofluorescence, following the principles described by Govindjee (1995).

Image analysis was conducted using ImageJ (Schneider et al., 2012) with the Image-based Tool for Counting Nuclei (ITCN) plugin. Fluorescence microscopy images were converted to 8-bit grayscale, and the inverted *LUT* function was applied to enhance contrast. The region of interest was selected, and chloroplasts were quantified based on pixel intensity using the ITCN plugin, with a width parameter of 13 pixels and automatic distance detection. The analysis was set to identify dark peaks, and the final output was expressed as the number of detected chloroplasts per pixel.

### 2.3 Protein extraction and digestion

Protein extraction was performed according to the protocol described by Hurkman and Tanaka (1986), with adjustments. Samples (100 mg) of grounded lyophilized plant material were homogenized in 800 µL of extraction buffer (0.7 M sucrose, 0.5 M Tris–HCl, pH 8.0, 0.1 M KCl, 50 mM EDTA, 2 mM PMSF, 2% β-mercaptoethanol, and 1% PVPP) and incubated under agitation at 4°C for 30 min. The homogenate was then mixed with an equal volume of Tris–HCl (pH 8.5)-saturated phenol and further agitated at 4°C for 30 min, followed by centrifugation (10,000 × *g*), for 30 min at 4°C. The supernatant was transferred to a new tube, and two additional washes were performed, using an equal volume of the extraction buffer; the first one, containing PVPP, and the second one, without PVPP, following the same previous agitation and centrifugation steps. Proteins were precipitated overnight at −20°C by adding five times the volume of 0.1 M ammonium acetate in methanol. After precipitation, samples were centrifuged (16,000 × *g*), for 30 min at 4°C and, sequentially, the pellet was washed twice with 0.1 M ammonium acetate in methanol, followed by a final wash with acetone (100%). Between each washing step, the samples were incubated at −20°C for 1 h, followed by centrifugation (16,000 × *g*), for 30 min at 4°C. The final protein pellet was dried in a desiccator at 4°C. The extracted proteins were solubilized in 400 µL of solubilization buffer [7 M urea, 2 M thiourea, 0.1% Triton X-100, and 10 mM dithiothreitol (DTT)]. Protein extracts were desalinized by Amicon^®^Ultra-0.5 mL 3K-NMWL filter devices (Millipore).

The protein concentration was determined using the Bradford assay (Bradford, 1976) and confirmed by sodium dodecyl sulfate-polyacrylamide gel electrophoresis (SDS-PAGE) (Laemmli, 1970). Ten micrograms of protein sample (1 µg µL^−1^) was denatured with 2.5 μL of 0.2% RapiGest SF (Waters) at 80°C for 15 min, reduced with 0.625 μL of 100 mM DTT (GE Healthcare) at 60°C for 30 min, and alkylated with 0.66 μL of 300 mM iodoacetamide (GE Healthcare) at room temperature (RT) for 30 min in the dark. Samples were then enzymatically digested with trypsin (Sequencing Grade Modified Trypsin, Promega) at a 1:100 (w/w) enzyme:protein ratio. After digestion, 1 μL of 5% trifluoroacetic acid was added to the digested mixture. The digested samples were then incubated at 37°C for 90 min to hydrolyze the RapiGest. After digestion, the mixture of peptides was centrifuged at 4°C at 16,000 × *g*, for 30 min. The supernatants were recovered and transferred to a vial and dried in the speedVac. The resulting peptides were suspended and desalinized with C18 ZipTips (Millipore, Billerica, MA, USA) following the manufacturer’s instructions and dried in the speedVac. The final volume of 50 μL was obtained by the addition of 0.1% formic acid water solution, to reach a concentration of 200 ng μL^−1^.

### 2.4 MS/MS analysis

Mass spectrometry analyses were performed using a nanoElute nanoflow chromatography system (Bruker, Bremen, Germany) coupled online to a timsTOF Pro mass spectrometer (Bruker). Peptides were separated using an Aurora 2 C18 trap column (1.6 µm, 250 mm × 75 µm, IonOpticks) under a nanoflow liquid chromatography system operating at 250 nL/min. A reverse-phase gradient was applied, using a 2%–95% gradient of solvent B [0.1% formic acid, 99.9% acetonitrile (v/v)]. The timsTOF Pro mass spectrometer was equipped with a CaptiveSpray ionization source (Bruker). The capillary transfer line temperature was set to 180°C, and ion accumulation was set to 123 ms. Ion mobility separation was achieved using an entrance potential ramp from −160 to −20 V over 123 s. Data acquisition was obtained using the parallel accumulation-serial fragmentation (PASEF) method, which enables simultaneous precursor ion accumulation and fragmentation. Precursor ions were initially identified through a full-scan tims-MS experiment covering an *m*/*z* range of 100–1,700. Singly charged precursors were excluded based on their position in the *m*/*z*-ion mobility plane, while precursors reaching an intensity threshold of 20,000 a.u. were dynamically excluded for 0.4 min to optimize fragmentation efficiency.

### 2.5 Bioinformatics and data processing

Mass spectrometry data were processed using MaxQuant v.2.4.0.0 (Tyanova et al., 2016a), employing spectral correlation against the *E. grandis* protein database v.2.0, available on the Phytozome platform (Goodstein et al., 2012). Fragment ion mass tolerance was set to 0.5 Da, and trypsin specificity was applied, allowing for up to two missed cleavage sites. Methionine oxidation and N-terminal acetylation were considered variable modifications and cysteine carbamidomethylation was set as a fixed modification. Peptide identification required a minimum length of seven amino acids, and both peptide and protein false discovery rates (FDRs) were controlled at 1%. At least one unique peptide was considered valid for protein inference. Proteomics data processing was conducted using Perseus software (Tyanova et al., 2016b). The protein matrix of each tissue was reduced by removing potential contaminants, proteins identified exclusively by a modification site, and reverse proteins. Only proteins detected in at least 50% of the samples within a given group were considered for downstream analysis. Protein abundance was inferred from the Total Ion Current (TIC) assigned to each protein and normalized by the sum of the TIC for all proteins in each sample using R studio. Then, proteins were log-transformed and data were processed using Pareto scaling with MetaboAnalyst 6.0 (Pang et al., 2024). Differential protein abundance was assessed using an unpaired, two-tailed Student’s *t*-test implemented in MetaboAnalyst. *p*-values were adjusted for multiple testing using the Benjamini–Hochberg FDR. Differentially abundant proteins were identified using volcano plots with thresholds of fold change > 2.0 and FDR-adjusted *p*-value < 0.05. The resulting lists of differentially abundant proteins for bark and xylem tissues are provided in **Supplementary Tables S1 and S2**, respectively. Gene Ontology (GO) enrichment analyses were performed using the DAPs defined by the same fold change (>2.0) and FDR criteria, comparing their GO term distributions against the complete proteome background of each tissue. Enrichment analyses were conducted in RStudio using the enrichplot package (Yu, 2025).

To investigate age- and tissue-dependent regulation of central metabolic pathways, curated representations of carbon metabolism (Boaretto et al., 2021) and the Calvin–Benson cycle (Michelet et al., 2013) were used as functional frameworks. For pathway-focused proteoform-level comparisons, normalized protein abundance values were compared between age groups using a two-tailed Student’s *t*-test assuming unequal variances (Welch’s *t*-test), applying a less stringent fold change threshold (>1.5) to capture moderate but biologically relevant abundance differences within metabolic pathways. Proteoform inference was conducted by mapping proteins associated with each pathway using *E. grandis* accessions from the Phytozome database. Subcellular localization predictions were obtained using the DeepLoc 2.0 web tool (Thumuluri et al., 2022) to support functional interpretation. In addition, the summed mean abundance of all identified proteoforms within each pathway was calculated to represent their relative contribution across tissues and developmental stages. Functional protein descriptions and associated biological processes were assigned using UniProtKB.

## 3 Results

### 3.1 Chloroplast distribution in bark and xylem across ages

**Figure 1:**
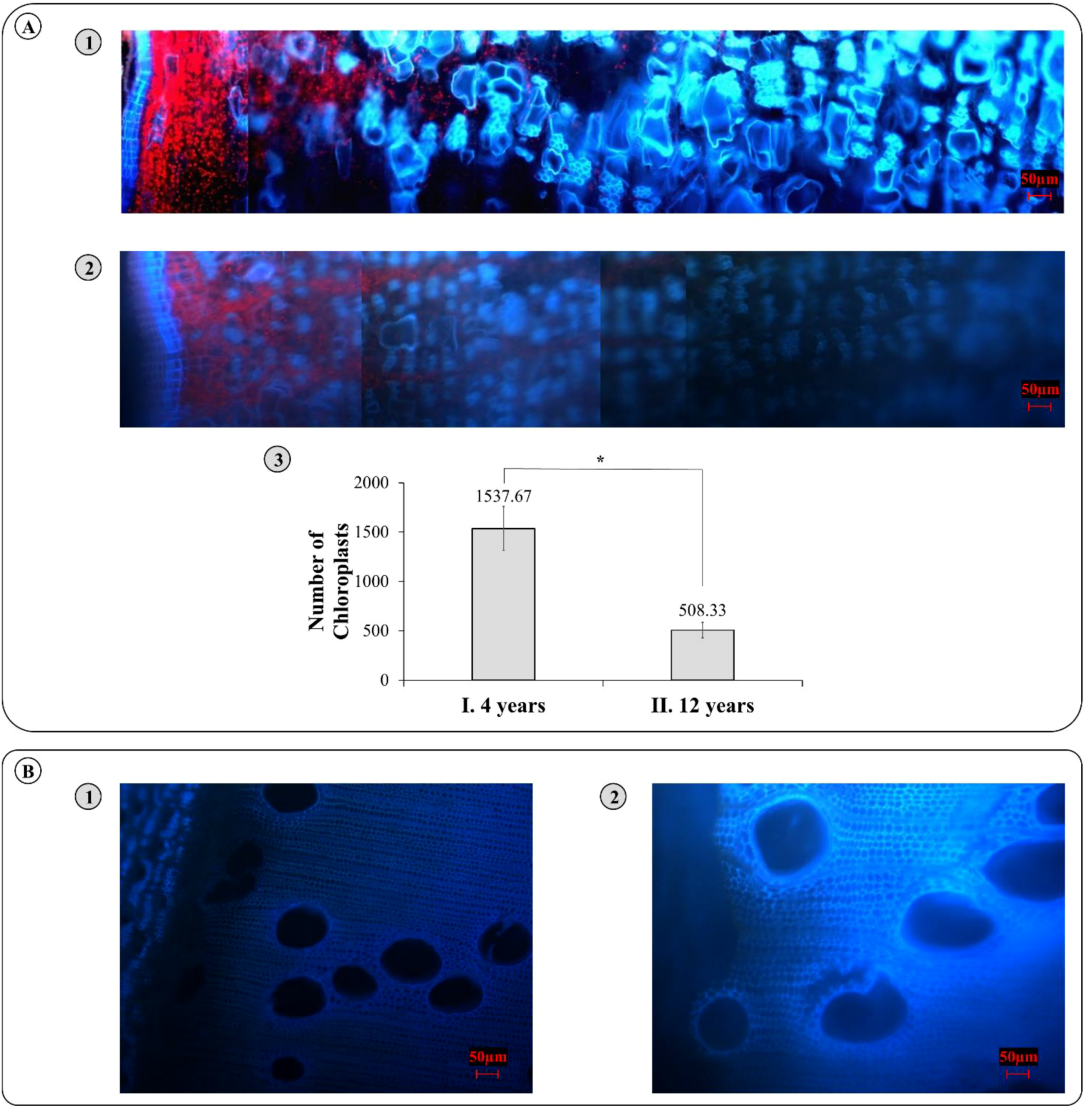
Fluorescence microscopy analysis of chloroplast abundance in the bark and xylem of *Eucalyptus grandis* at 4 and 12 years of age. **(A)** Representative fluorescence microscopy images of the bark highlighting chloroplasts. **(A1)** Bark from 4-year-old plants and **(A2)** bark from 12-year-old plants. **(A3)** Quantification of chloroplast abundance in bark. Bars represent mean ± standard deviation (*n* = 3 biological replicates) and * represents statistical difference by Student’s *t*-test (*p*-value = 0.0329). **(B)** Fluorescence microscopy images of xylem tissue, showing the absence of chloroplasts in both **(B1)** 4-year-old and **(B2)** 12-year-old plants.

Fluorescence microscopy was performed on *E. grandis* tissues from 4-year-old and 12-year-old trees to analyze the distribution and abundance of chloroplasts. For that, three biological replicates of transverse sections from bark (**Figure 1A**) to xylem (**Figure 1B**) were analyzed and the number of chloroplasts identified by autofluorescence was evaluated. These data show that, in both age groups, chloroplasts were found in the bark, but not in xylem tissues. Furthermore, an average of 1,538 and 508 chloroplasts were detected in 4-year-old and 12-year-old plants, respectively (**Figure 1A3**). The abundance of chloroplasts varied significantly (*p-*value = 0.0329) between the two developmental stages. These quantitative differences in chloroplast abundance provide an anatomical basis for the age-dependent changes in bark metabolism explored in the subsequent proteomic analyses.

### 3.2 Comparison between the age-dependent bark and xylem of *E. grandis*

Proteomic analysis of xylem tissue in 4-year-old *E. grandis* trees identified 2,593 proteins, whereas 2,652 proteins were detected in 12-year-old trees. Collectively, 3,113 non-redundant xylem proteins were identified, with 532 exhibiting statistically significant differential abundance (*t*-test, *p* < 0.05). Similarly, bark tissue analysis revealed 3,076 proteins in 4-year-old trees and 2,708 proteins in 12-year-old trees. A total of 3,446 non-redundant bark proteins were detected, of which 348 showed significant differences in abundance between age groups (*t*-test, *p* < 0.05) (see **Supplementary Tables 1, 2**).

**Figure 2:**
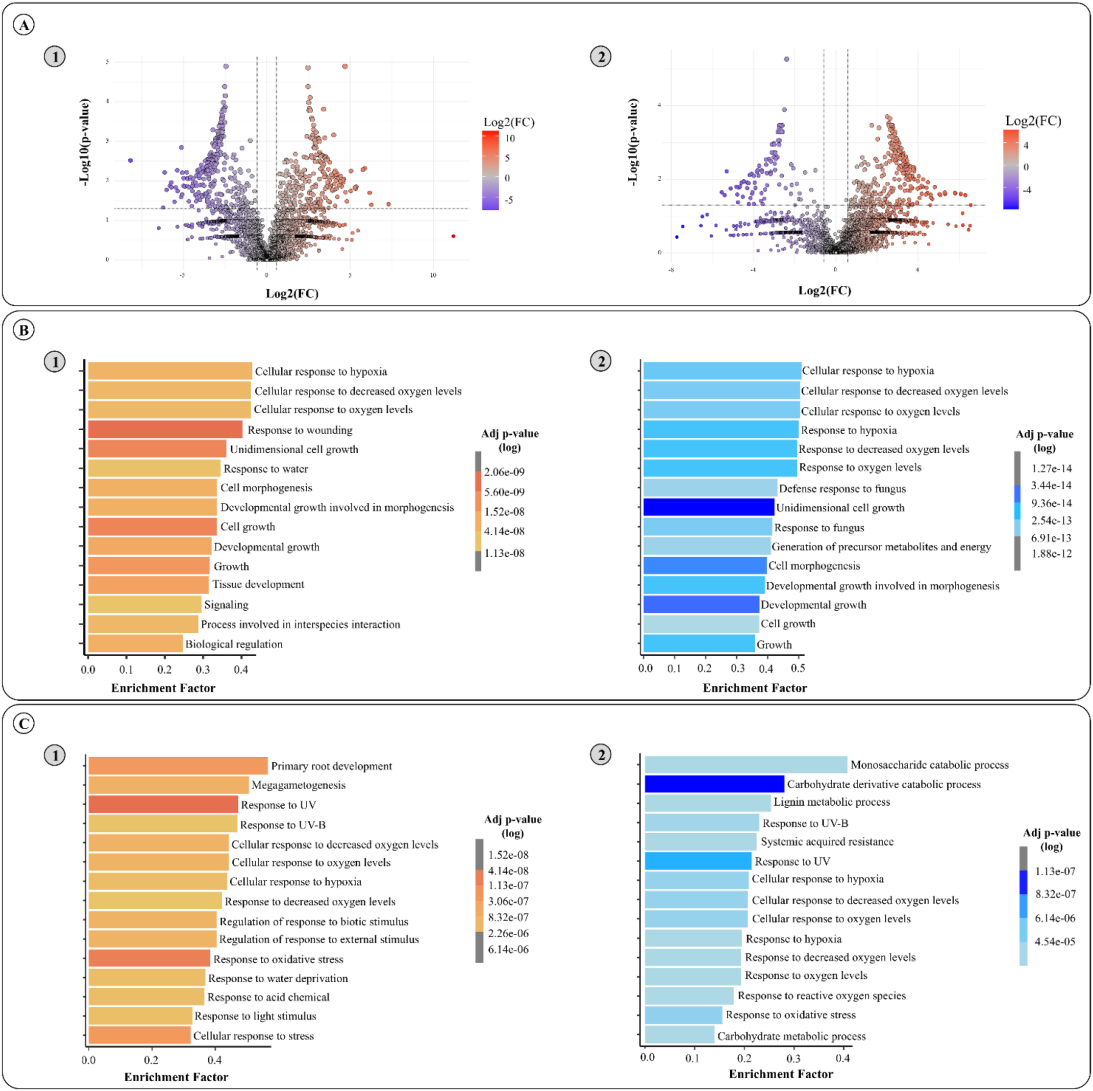
Proteomic analysis and functional enrichment of the top 15 GO terms enriched in both xylem and bark tissues from *Eucalyptus grandis* at 4 and 12 years of age. **(A)** Volcano plots comparing differential protein abundance (log2 fold change) between 4-year-old and 12-year-old plants in **(A1)** xylem and **(A2)** bark tissues. Significant proteins are indicated by adjusted *p*-value thresholds (colored points). **(B)** Functional enrichment analysis of differentially abundant proteins in **(B1)** xylem and **(B2)** bark tissues at 4 years of age. The *x*-axis represents the enrichment factor, and the color gradient corresponds to adjusted *p*-values (log scale). **(C)** Functional enrichment analysis of differentially abundant proteins in **(C1)** xylem and **(C2)** bark tissues at 12 years of age. Like **(B)**, the *x*-axis shows the enrichment factor, and the color gradient reflects adjusted *p*-values.

Differentially abundant proteins between 4- and 12-year-old trees were identified separately for bark and for xylem (fold change > 2.0; Student’s *t*-test, *p* < 0.05). The results show significant differences between ages within each tissue. In the xylem, 230 proteins were more abundant at 4 years, while 282 proteins were more abundant in 12-year-old trees. In the bark, 250 proteins were more abundant in 4-year-old trees, whereas 88 proteins were more abundant at 12 years (**Figures 2A1, A2**). Differentially abundant proteins were contrasted with their associated GO terms against all identified proteins in each tissue, leading to an enrichment analysis. The enrichment analysis (**Figures 2B, C**) highlights the 15 most enriched biological processes, displayed alongside their gradient of statistical significance (adjusted *p*-value).

**Figure 3:**
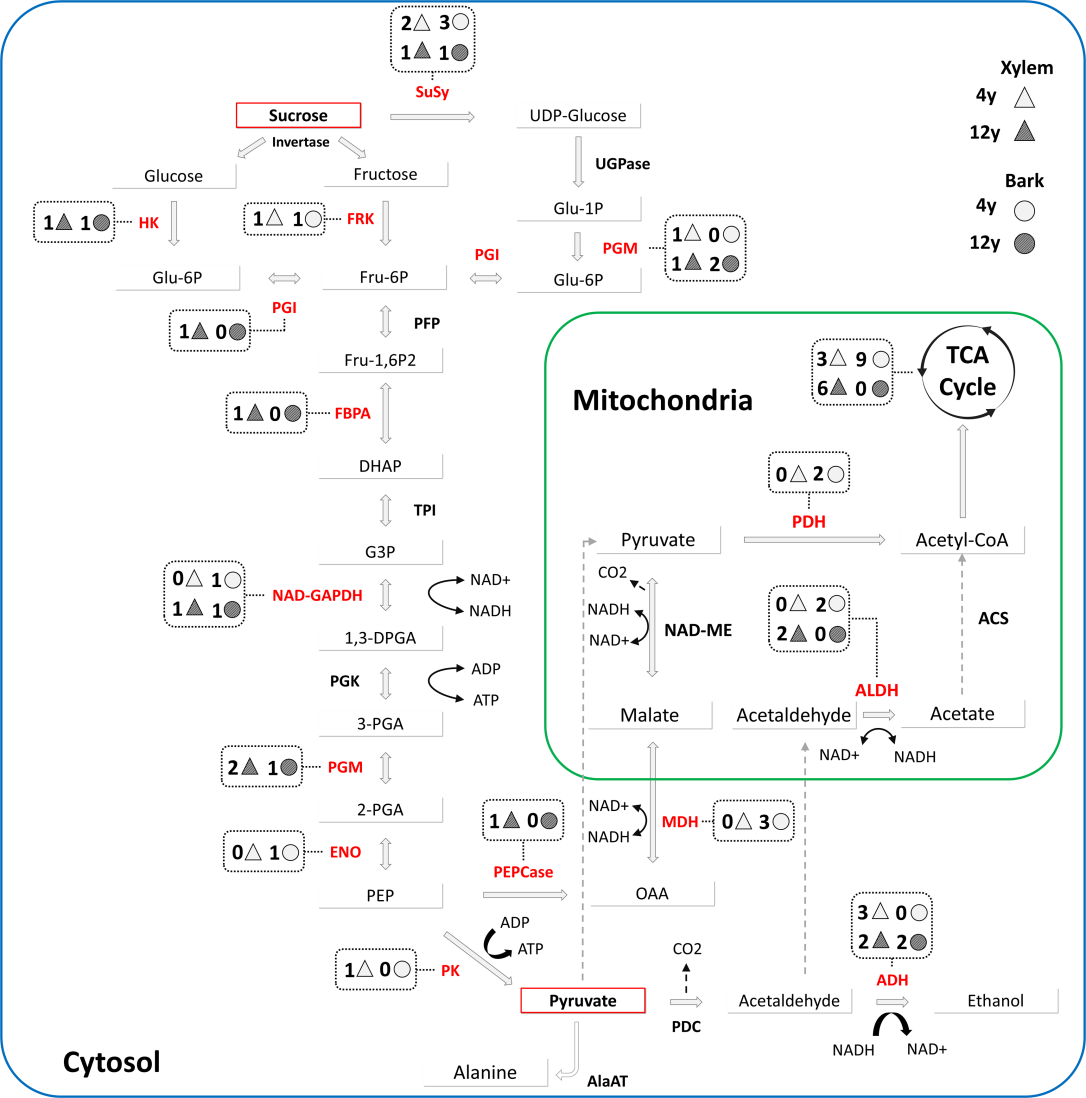
Schematic representation of enzymes involved in primary carbon metabolism in *Eucalyptus grandis* xylem and bark tissues. The diagram illustrates enzymes participating in glucose metabolism, fermentation, and the tricarboxylic acid cycle. Enzymes shown in red are those associated with significant functional terms identified in the proteomic analysis. Numbers within shapes represent the proteoforms with differential abundance (fold change > 1.5, *p*-value < 0.05) for each enzyme. Circles and triangles indicate data from bark and xylem tissues, respectively. The empty and filled forms represent 4-year-old and 12-year-old plants, respectively.

In general, the enrichment analysis revealed distinct biological processes in either tissue or age. In the xylem of 4-year-old plants, processes related to oxygen availability were most represented, with “cellular response to hypoxia” and “response to decreased oxygen levels”, followed by growth and developmental pathways, including “cell morphogenesis” and “developmental growth involved in morphogenesis”, indicating active cell proliferation and differentiation. Processes related to oxygen availability, such as “cellular response to hypoxia” and “response to decreased oxygen levels”, were also enriched. Similarly, 4-year-old bark not only exhibited enrichment in hypoxia-related pathways but also showed the term “generation of precursor metabolites and energy”, which aligns with the presence of chloroplasts in this tissue. Consistent with this enrichment, 10 proteoforms from the TCA cycle were more abundant in 4-year-old bark compared with 12-year-old bark (**Figure 3**), supporting enhanced generation of precursor metabolites and energy in younger bark. Additionally, bark displayed response to other environmental stimuli, like response to fungus. In contrast, in older tissues while hypoxia-related processes remain enriched, there is an increased presence of terms associated to plant homeostasis in xylem and carbohydrate metabolisms in bark. Together, these findings highlight dynamic metabolic differences between young and mature tissues, reinforcing the distinct functional roles of xylem and bark over time.

### 3.3 Carbon metabolism and Calvin–Benson cycle activity in *E. grandis*

To study the carbon metabolism of *E. grandis* and show the differences between age-specific bark and xylem tissues, the proteoforms related to glycolysis, fermentation, TCA cycle, and Calvin–Benson cycle (Calvin and Benson, 1948) were highlighted (**Table 1**) and compared. **Table 1** provides a detailed overview of enzymes involved in these pathways, emphasizing their distribution across tissues. Notably, all enzymes in these metabolic processes were identified, with some proteoforms being tissue-specific. For instance, Malic Enzyme (Eucgr.C00609.1.p) and Fumarase (Eucgr.H01081.1.p) were exclusively detected in the xylem. Malic Enzyme plays a role in converting malate to pyruvate, linking glycolysis and the TCA cycle by supplying pyruvate for energy production. Fumarase catalyzes the reversible hydration of fumarate to malate in the TCA cycle, maintaining the metabolic flux. Their absence in other tissues may be due to biological specialization or due to its very low abundance for detection. A noteworthy observation concerns ADH, for which 20 proteoforms were identified, with 6 exhibiting differential abundance between the age groups, within the same tissue, suggesting age-related metabolic adaptations for alcoholic fermentation. For the photosynthesis-related enzymes, proteins of the photosystems I and II did not show significant differences, but eight enzymes within the Calvin–Benson cycle displayed proteoforms that were more abundant in 4-year-old plants; in particular, proteoforms of ribulose-1,5-bisphosphate carboxylase/oxygenase (RuBisCO) (Eucgr.J01234.1.p, Eucgr.J02030.1.p, and Eucgr.J02030.1.p) demonstrated fold changes exceeding 4. The relative abundance of all proteoforms is depicted in **Supplementary Figure 2**, revealing a coherent pattern in which chloroplast-related and Calvin–Benson cycle enzymes are more strongly represented in younger tissues, supporting a higher potential for local carbon fixation in juvenile bark. These findings collectively suggest distinct metabolic profiles between tissues and developmental stages in *E. grandis*, with younger plants exhibiting an enhanced proteomic profile of chloroplastic activity and carbon assimilation capabilities.

**Table 1:** List of proteoforms identified by LC-MS/MS for enzymes involved in glycolysis, fermentation, the TCA cycle, and the Calvin-Benson cycle.

Access^1^	Description^2^	Xylem^3^		Bark^4^	
		FC	*p*-value	FC	*p*-value
Sucrose synthase (SuSy)					
Eucgr.C00769.1.p*	Sucrose synthase/Uridine diphosphoglucose-fructose glucosyltransferase	2.1	0.048	11.0	0.142
Eucgr.C03199.1.p*	Sucrose synthase/Uridine diphosphoglucose-fructose glucosyltransferase	2.1	0.024	6.3	0.039
Eucgr.C01715.1.p*	Sucrose-phosphate synthase	0.1	0.043	0.2	0.019
Eucgr.E03524.1.p	Sucrose-phosphate synthase/UDP-glucose-fructose-phosphate glucosyltransferase	0.6	0.193	1.5	0.385
Eucgr.J01640.1.p	SUCROSE SYNTHASE 5	–	–	6.1	0.003
Eucgr.K02305.1.p	SUCROSE SYNTHASE 6	–	–	5.4	0.002
UDP-glucose pyrophosphorylase (UGPase)					
Eucgr.F02905.1.p	UDP-GLUCOSE PYROPHOSPHORYLASE	0.7	0.122	0.9	0.811
Eucgr.D01117.1.p	UDP-glucose pyrophosphorylase	0.3	0.182	1.7	0.352
Fructokinase (FRK)					
Eucgr.A00095.1.p*	Fructokinase PROTEIN-RELATED	1.5	0.035	3.3	0.000
Hexokinase (HK)					
Eucgr.C00559.1.p*	Hexokinase/Hexokinase type IV (glucokinase)	0.2	0.003	0.1	0.009
Eucgr.C03728.1.p	Hexokinase/Hexokinase type IV (glucokinase)	1.3	0.217	1.2	0.467
Eucgr.F01647.1.p	HEXOKINASE-LIKE 1 PROTEIN	3.1	0.182	3.8	0.208
Glucose 6 phosphate isomerase (PGI)					
Eucgr.F01561.1.p*	GLUCOSE-6-PHOSPHATE ISOMERASE, CYTOSOLIC	0.2	0.024	0.6	0.054
Pyrophosphate fructose 6 phosphate (PFP)					
Eucgr.F01823.1.p	Diphosphate--fructose-6-phosphate 1-phosphotransferase/Pyrophosphate-dependent 6-phosphofructose-1-kinase	1.4	0.098	1.4	0.305
Eucgr.F03611.1.p	Diphosphate--fructose-6-phosphate 1-phosphotransferase/Pyrophosphate-dependent 6-phosphofructose-1-kinase	0.2	0.072	0.3	0.191
Fructose bisphosphate aldolase (FBPA)					
Eucgr.A01538.1.p	Fructose-bisphosphate aldolase/Fructose-1,6-bisphosphate triosephosphate-lyase	1.1	0.069	1.0	0.880
Eucgr.B02864.1.p	FRUCTOSE-BISPHOSPHATE ALDOLASE 3, CHLOROPLASTIC-RELATED	–	–	0.7	0.096
Eucgr.G01726.3.p	FRUCTOSE-BISPHOSPHATE ALDOLASE 2, CHLOROPLASTIC-RELATED	–	–	3.4	0.139
Eucgr.I01326.1.p	FRUCTOSE-BISPHOSPHATE ALDOLASE 2, CHLOROPLASTIC-RELATED	–	–	3.4	0.105
Eucgr.K02073.1.p*	FRUCTOSE-BISPHOSPHATE ALDOLASE	0.5	0.002	0.9	0.345
Eucgr.F02017.1.p	FRUCTOSE-BISPHOSPHATE ALDOLASE-RELATED	–	–	3.1	0.062
Triosephosphate isomerase (TPI)					
Eucgr.I01374.1.p*	TRIOSEPHOSPHATE ISOMERASE, CHLOROPLASTIC	–	–	1.6	0.035
Eucgr.J00008.1.p	Triose-phosphate isomerase/Triosephosphate mutase	0.9	0.512	0.6	0.062
Eucgr.J02049.1.p	Triose-phosphate isomerase/Triosephosphate mutase	1.2	0.134	1.5	0.157
NAD-dependent glyceraldehyde 3 phosphate dehydrogenase (NAD-GAPDH)					
Eucgr.C01646.1.p	Glyceraldehyde-3-phosphate dehydrogenase (NADP(+))/Triosephosphate dehydrogenase	0.2	0.073	0.1	0.023
Eucgr.C01947.1.p	Glyceraldehyde-3-phosphate dehydrogenase (NADP(+))/Triosephosphate dehydrogenase	0.1	0.197	0.6	0.313
Eucgr.B00144.1.p*	Glyceraldehyde-3-phosphate dehydrogenase (phosphorylating)/Triosephosphate dehydrogenase	0.3	0.024	0.7	0.053
Eucgr.C02594.1.p*	NADP-DEPENDENT GLYCERALDEHYDE-3-PHOSPHATE DEHYDROGENASE	–	–	3.3	0.017
Phosphoglycerate kinase (PGK)					
Eucgr.F01476.1.p*	PHOSPHOGLYCERATE KINASE 1, CHLOROPLASTIC-RELATED	–	–	1.8	0.024
Eucgr.F04463.1.p	PHOSPHOGLYCERATE KINASE	1.0	0.769	1.0	0.977
Phosphoglycerate mutase (PGM)					
Eucgr.J01450.1.p	COFACTOR-INDEPENDENT PHOSPHOGLYCERATE MUTASE	1.2	0.820	1.1	0.822
Eucgr.I00964.1.p	PHOSPHOGLYCERATE MUTASE	2.6	0.336	–	–
Eucgr.A00868.1.p	Phosphoglycerate mutase (2,3-diphosphoglycerate-dependent)/Phosphoglyceromutase	0.8	0.103	1.3	0.162
Eucgr.K01314.1.p*	Phosphoglycerate mutase (2,3-diphosphoglycerate-dependent)/Phosphoglyceromutase	0.0	0.007	0.4	0.019
Eucgr.E01587.1.p*	Predicted phosphoglycerate mutase	0.2	0.005	8.4	0.315
Enolase (ENO)					
Eucgr.J01952.1.p*	Phosphopyruvate hydratase/Enolase	1.1	0.739	2.7	0.008
Phosphoenolpyruvate carboxylase (PEPCase)					
Eucgr.A01915.1.p*	Phosphoenolpyruvate carboxylase/Phosphoenolpyruvic carboxylase	0.4	0.003	0.9	0.573
Eucgr.F01229.1.p	Phosphoenolpyruvate carboxylase/Phosphoenolpyruvic carboxylase	0.9	0.661	1.2	0.423
Eucgr.B03067.1.p	PHOSPHOENOLPYRUVATE CARBOXYLASE 4	0.1	0.107	0.8	0.326
Pyruvate kinase (PK)					
Eucgr.G02720.1.p	PYRUVATE KINASE	0.9	0.735	1.2	0.290
Eucgr.K01637.1.p*	PYRUVATE KINASE	5.0	0.000	2.7	0.139
Eucgr.K02074.1.p	PYRUVATE KINASE	1.2	0.067	1.0	0.934
Eucgr.J00327.1.p	PYRUVATE KINASE-RELATED	0.9	0.670	0.9	0.498
Eucgr.J01749.1.p	PYRUVATE KINASE-RELATED	1.4	0.287	0.5	0.068
Eucgr.B03586.1.p	PYRUVATE KINASE-RELATED	–	–	2.1	0.430
Malate dehydrogenase (MDH)					
Eucgr.H03047.1.p	MALATE DEHYDROGENASE	1.2	0.417	1.3	0.177
Eucgr.J01037.1.p*	Malate dehydrogenase (NADP(+))/NADP-linked malate dehydrogenase	0.2	0.067	3.4	0.007
Eucgr.C01003.1.p	Malate dehydrogenase (oxaloacetate-decarboxylating) (NADP(+))/Pyruvic-malic carboxylase	1.2	0.590	1.7	0.118
Eucgr.H02358.1.p	MALATE DEHYDROGENASE, GLYOXYSOMAL-RELATED	0.7	0.351	0.5	0.173
Eucgr.E01596.1.p*	MALATE DEHYDROGENASE	–	–	10.1	0.049
Eucgr.H02364.1.p	MALATE DEHYDROGENASE, GLYOXYSOMAL-RELATED	–	–	4.0	0.215
Eucgr.D02251.1.p*	Malate dehydrogenase (decarboxylating)/Pyruvic-malic carboxylase (mitochondrion)	0.9	0.355	1.7	0.002
Eucgr.E01713.1.p	Malate dehydrogenase (decarboxylating)/Pyruvic-malic carboxylase (mitochondrion)	1.1	0.713	1.3	0.494
Eucgr.F01209.1.p	Malate dehydrogenase/Malic dehydrogenase (mitochondrion)	1.5	0.051	0.4	0.390
Eucgr.F03251.1.p	Malate dehydrogenase/Malic dehydrogenase (mitochondrion)	3.3	0.054	0.5	0.453
Pyruvate dehydrogenase (PDH)					
Eucgr.F02506.1.p*	PYRUVATE DEHYDROGENASE E1 COMPONENT SUBUNIT BETA, MITOCHONDRIAL	0.7	0.122	2.4	0.027
Eucgr.B03379.1.p*	PYRUVATE DEHYDROGENASE E1 COMPONENT, ALPHA SUBUNIT BACTERIAL AND ORGANELLAR	1.4	0.059	1.9	0.045
Eucgr.H04122.1.p	[Pyruvate dehydrogenase (acetyl-transferring)] kinase/Pyruvate dehydrogenase kinase (phosphorylating)	–	–	3.3	0.186
Alanine aminotransferase (AlaAT)					
Eucgr.H04825.1.p	ALANINE--GLYOXYLATE AMINOTRANSFERASE 2, MITOCHONDRIAL	3.4	0.191	1.3	0.472
Eucgr.K01268.1.p	GLYOXYLATE AMINOTRANSFERASE 2 HOMOLOG 2, MITOCHONDRIAL-RELATED	1.2	0.844	–	–
Alcohol dehydrogenase (ADH)					
Eucgr.I00224.1.p	ALCOHOL DEHYDROGENASE CLASS-P	0.8	0.334	0.9	0.894
Eucgr.I00224.2.p*	ALCOHOL DEHYDROGENASE CLASS-P	0.2	0.024	0.5	0.071
Eucgr.I00224.3.p*	ALCOHOL DEHYDROGENASE CLASS-P	0.5	0.002	0.6	0.033
Eucgr.A02910.1.p	ALCOHOL DEHYDROGENASE RELATED	1.1	0.656	0.8	0.311
Eucgr.D01327.1.p*	ALCOHOL DEHYDROGENASE RELATED	3.6	0.009	3.2	0.184
Eucgr.E01107.1.p	ALCOHOL DEHYDROGENASE RELATED	0.7	0.291	0.9	0.893
Eucgr.F02744.1.p	ALCOHOL DEHYDROGENASE RELATED	0.8	0.379	1.5	0.249
Eucgr.F02744.3.p	ALCOHOL DEHYDROGENASE RELATED	0.8	0.151	1.0	0.743
Eucgr.H04903.3.p	ALCOHOL DEHYDROGENASE RELATED	1.4	0.317	2.5	0.055
Eucgr.H04950.1.p	ALCOHOL DEHYDROGENASE RELATED	1.8	0.065	0.9	0.860
Eucgr.I01803.2.p	ALCOHOL DEHYDROGENASE RELATED	17.1	0.256	0.6	0.709
Eucgr.K00070.1.p	ALCOHOL DEHYDROGENASE RELATED	0.9	0.912	1.2	0.505
Eucgr.K00071.2.p	ALCOHOL DEHYDROGENASE RELATED	1.8	0.460	0.9	0.908
Eucgr.K00793.4.p*	ALCOHOL DEHYDROGENASE RELATED	1.7	0.037	7.2	0.124
Eucgr.K00795.1.p	ALCOHOL DEHYDROGENASE RELATED	1.7	0.403	–	–
Eucgr.K02331.2.p*	ALCOHOL DEHYDROGENASE-LIKE 6	2.3	0.012	5.9	0.277
Eucgr.E01104.1.p	ALCOHOL DEHYDROGENASE RELATED	–	–	4.7	0.244
Eucgr.E01117.5.p*	ALCOHOL DEHYDROGENASE RELATED	–	–	0.1	0.040
Eucgr.E01119.5.p	ALCOHOL DEHYDROGENASE RELATED	–	–	0.7	0.159
Eucgr.I01800.1.p	ALCOHOL DEHYDROGENASE RELATED	–	–	0.1	0.168
Malic enzyme (NAD-ME)					
Eucgr.C00609.1.p	NADP-DEPENDENT MALIC ENZYME 2-RELATED	6.1	0.111	–	–
Phosphoglucomutase (PGM)					
Eucgr.K00185.2.p	Phosphoglucomutase (glucose-cofactor)/Glucose-1-phosphate phosphotransferase	0.1	0.139	0.9	0.695
Eucgr.J01084.1.p*	Phosphoglucomutase (alpha-D-glucose-1,6-bisphosphate-dependent)	0.3	0.008	0.5	0.018
Eucgr.G02157.1.p*	PHOSPHOGLUCOMUTASE, CYTOPLASMIC 1-RELATED	0.9	0.317	0.6	0.028
Eucgr.B02942.2.p*	PHOSPHOGLUCOMUTASE, CYTOPLASMIC 1-RELATED	1.9	0.021	1.4	0.203
Pyruvate decarboxylase (PDC)					
Eucgr.A00549.1.p	Pyruvate decarboxylase/Pyruvic decarboxylase	1.5	0.117	0.9	0.732
NAD-aldehyde dehydrogenase (ALDH)					
Eucgr.B00357.1.p*	ALDEHYDE DEHYDROGENASE FAMILY 2 MEMBER B7, MITOCHONDRIAL	0.2	0.004	1.1	0.561
Eucgr.B03349.2.p*	ALDEHYDE DEHYDROGENASE FAMILY 2 MEMBER B7, MITOCHONDRIAL	0.1	0.005	1.9	0.248
Eucgr.C03853.3.p	ALDEHYDE DEHYDROGENASE FAMILY 2 MEMBER C4	–	–	2.7	0.484
Eucgr.F03800.3.p*	ALDEHYDE DEHYDROGENASE FAMILY 3 MEMBER I1	1.0	0.923	5.1	0.003
Eucgr.H04040.3.p	ALDEHYDE DEHYDROGENASE-LIKE PROTEIN YHR039C-RELATED	–	–	3.0	0.182
Eucgr.H05081.2.p	ALDEHYDE DEHYDROGENASE FAMILY 3 MEMBER I1, CHLOROPLASTIC	–	–	9.2	0.058
Eucgr.I01821.1.p	ALDEHYDE DEHYDROGENASE FAMILY 2 MEMBER B7, MITOCHONDRIAL	–	–	0.5	0.141
Eucgr.K02858.2.p*	ALDEHYDE DEHYDROGENASE FAMILY 2 MEMBER B7, MITOCHONDRIAL	1.2	0.260	2.6	0.005
Eucgr.C03859.1.p	ALDEHYDE DEHYDROGENASE FAMILY 2 MEMBER C4	0.2	0.089	–	–
Acetyl-CoA synthetase (ACS)					
Eucgr.K02641.2.p	Acetyl-coenzyme A synthetase N-terminus (ACAS_N)	6.3	0.084	–	–
Eucgr.J02955.1.p	ACYL-COA SYNTHETASE FAMILY MEMBER 3, MITOCHONDRIAL	–	–	7.6	0.305
ATP citrate synthase (ACLY)					
Eucgr.G02039.3.p*	ATP citrate synthase/Citric cleavage enzyme	2.2	0.004	1.8	0.235
Eucgr.H04045.2.p*	ATP citrate synthase/Citric cleavage enzyme	2.9	0.006	4.5	0.086
Eucgr.I00055.1.p*	ATP citrate synthase/Citric cleavage enzyme	2.0	0.000	5.1	0.045
Eucgr.A01955.2.p*	Citrate (Si)-synthase/Citrate oxaloacetate-lyase ((pro-3S)-CH(2)COO->acetyl-CoA)	0.1	0.008	1.2	0.761
Eucgr.G03412.1.p*	Citrate (Si)-synthase/Citrate oxaloacetate-lyase ((pro-3S)-CH(2)COO->acetyl-CoA)	0.5	0.004	1.0	0.964
Aconitase hydratase (ACO)					
Eucgr.C03206.1.p	Aconitate hydratase/Citrate(isocitrate) hydro-lyase	0.9	0.877	0.0	0.346
Eucgr.I02307.1.p	Aconitate hydratase/Citrate(isocitrate) hydro-lyase	1.0	0.913	0.8	0.054
Eucgr.A01129.1.p	ACONITATE HYDRATASE 3, MITOCHONDRIAL	0.9	0.444	1.3	0.389
NAD-dependent isocitrate dehydrogenase (IDH)					
Eucgr.A01135.1.p	Isocitrate dehydrogenase (NAD(+))/Nicotinamide adenine dinucleotide isocitrate dehydrogenase	0.7	0.043	1.3	0.332
Eucgr.I01510.2.p	Isocitrate dehydrogenase (NAD(+))/Nicotinamide adenine dinucleotide isocitrate dehydrogenase	0.7	0.459	1.1	0.838
Eucgr.I02232.1.p	Isocitrate dehydrogenase (NAD(+))/Nicotinamide adenine dinucleotide isocitrate dehydrogenase	0.6	0.115	0.6	0.670
Eucgr.F02901.1.p	Isocitrate dehydrogenase (NADP(+))/oxalosuccinate carboxylase	0.9	0.321	1.5	0.155
Eucgr.J02223.1.p	Isocitrate dehydrogenase (NADP(+))/oxalosuccinate carboxylase	0.8	0.498	1.2	0.318
Oxoglutarate dehydrogenase (OGDH)					
Eucgr.G01375.1.p	Oxoglutarate dehydrogenase (succinyl-transferring)/Oxoglutarate dehydrogenase (lipoamide)	0.7	0.082	1.2	0.369
Succinyl-CoA ligase (SUCL)					
Eucgr.J00864.1.p	Succinate--CoA ligase (ADP-forming)/Succinyl-CoA synthetase (ADP-forming)	1.5	0.428	2.8	0.167
Eucgr.I00009.1.p	Succinate-semialdehyde dehydrogenase (acetylating)/Succinyl-CoA reductase	1.2	0.620	1.2	0.549
Eucgr.H00143.1.p*	SUCCINYL-COA SYNTHETASE BETA CHAIN	0.6	0.034	1.4	0.144
Eucgr.H00643.1.p	Succinate--CoA ligase (ADP-forming)/Succinyl-CoA synthetase (ADP-forming)	–	–	1.3	0.743
Succinate dehydrogenase (SDH)					
Eucgr.J00592.1.p	Succinate dehydrogenase (quinone)/Succinic dehydrogenase	0.1	0.132	0.0	0.320
Eucgr.J00347.1.p	SUCCINATE DEHYDROGENASE [UBIQUINONE] IRON-SULFUR SUBUNIT, MITOCHONDRIAL	1.1	0.714	1.4	0.413
Eucgr.I02452.1.p	SUCCINATE DEHYDROGENASE 2 FLAVOPROTEIN SUBUNIT	1.1	0.607	1.5	0.095
Eucgr.F04474.1.p*	SUCCINATE-SEMIALDEHYDE DEHYDROGENASE, MITOCHONDRIAL	0.6	0.042	1.7	0.039
Fumarase (FUM)					
Eucgr.H01081.1.p*	Fumarate hydratase/Fumarase	0.5	0.035	–	–
Photosystems I and II					
Eucgr.A00264.1.p	Photosystem II protein (PSII)	–	–	1.2	0.561
Eucgr.B01088.1.p	PHOTOSYSTEM I REACTION CENTER SUBUNIT II-1, CHLOROPLASTIC-RELATED	–	–	3.9	0.074
Eucgr.B02162.1.p	PHOTOSYSTEM I REACTION CENTER SUBUNIT IV A, CHLOROPLASTIC-RELATED	–	–	3.3	0.060
Eucgr.B03692.2.p	PHOTOSYSTEM II 5 KDA PROTEIN, CHLOROPLASTIC	–	–	0.8	0.747
Eucgr.F03245.1.p	Photosystem II 10 kDa phosphoprotein (PsbH)	–	–	0.1	0.112
Eucgr.F03789.1.p	PHOTOSYSTEM II 22 KDA PROTEIN, CHLOROPLASTIC	–	–	10.8	0.153
Eucgr.H00828.1.p	PHOTOSYSTEM I REACTION CENTER SUBUNIT IV A, CHLOROPLASTIC-RELATED	–	–	1.9	0.226
Eucgr.K00389.1.p	PHOTOSYSTEM I REACTION CENTER SUBUNIT III, CHLOROPLASTIC	–	–	2.6	0.174
Eucgr.K02362.3.p	PHOTOSYSTEM I REACTION CENTER SUBUNIT XI, CHLOROPLASTIC	–	–	3.7	0.203
Ribulose bisphosphate carboxylase oxygenase (RuBisCO)					
Eucgr.B02310.1.p	RIBULOSE BISPHOSPHATE CARBOXYLASE/OXYGENASE ACTIVASE, CHLOROPLASTIC	–	–	10.2	0.141
Eucgr.J01234.1.p*	RIBULOSE BISPHOSPHATE CARBOXYLASE/OXYGENASE ACTIVASE, CHLOROPLASTIC	–	–	4.1	0.000
Eucgr.J01502.1.p	Ribulose-bisphosphate carboxylase/RuBP carboxylase	–	–	2.0	0.133
Eucgr.J01502.2.p	Ribulose-bisphosphate carboxylase/RuBP carboxylase	–	–	2.1	0.194
Eucgr.J02030.1.p*	RIBULOSE BISPHOSPHATE CARBOXYLASE/OXYGENASE ACTIVASE, CHLOROPLASTIC	–	–	7.0	0.004
Eucgr.J02030.1.p*	RIBULOSE BISPHOSPHATE CARBOXYLASE/OXYGENASE ACTIVASE, CHLOROPLASTIC	–	–	5.7	0.033
Glyceraldehyde-3-phosphate dehydrogenase (GAPDH)					
Eucgr.F01793.1.p*	Glyceraldehyde-3-phosphate dehydrogenase (NADP(+)) (phosphorylating)	–	–	3.3	0.002
Eucgr.F04466.1.p	GLYCERALDEHYDE 3-PHOSPHATE DEHYDROGENASE-	–	–	1.8	0.072
Eucgr.I01564.1.p*	Glyceraldehyde-3-phosphate dehydrogenase (NADP(+)) (phosphorylating)	–	–	2.8	0.011
Phosphoribulokinase (PRK)					
Eucgr.E01261.2.p.1.p*	Phosphoribulokinase	–	–	3.6	0.003
Transketolase (TKL)					
Eucgr.D02466.1.p	Transketolase/Glycoaldehyde transferase	–	–	3.5	0.155
Eucgr.D02467.1.p*	Transketolase/Glycoaldehyde transferase/Transaldolase/Glycerone transferase	–	–	3.2	0.002
Ribulose-5-phosphate-3-epimerase (RPE)					
Eucgr.B00532.5.p	Ribulose-phosphate 3-epimerase/Xylulose phosphate 3-epimerase	–	–	1.6	0.381
Fructose-1.6-bisphosphatase (FBPase)					
Eucgr.B02755.1.p*	FRUCTOSE-1,6-BISPHOSPHATASE, CHLOROPLASTIC	–	–	2.5	0.009
Eucgr.F02711.1.p*	FRUCTOSE-1,6-BISPHOSPHATASE-RELATED	–	–	3.1	0.001
Eucgr.I02616.1.p	FRUCTOSE-1,6-BISPHOSPHATASE-RELATED	–	–	0.4	0.062
Sedoheptulose bisphosphatase (SBPase)					
Eucgr.J00242.1.p*	Sedoheptulose-bisphosphatase/Sedoheptulose-1,7-bisphosphatase	–	–	4.3	0.015

^1^Protein IDs from the Phytozome database.

^2^Protein description from the Phytozome database.

^3^Data from xylem tissue. Fold change (FC) = 4-year/12-year relative abundance.

^4^Data from bark tissue. Fold change (FC) = 4-year/12-year relative abundance.

*Proteoform considered differentially abundant.

*p*-values < 0.05 were considered statistically significant according to Student’s *t*-test.

**Figure 4:**
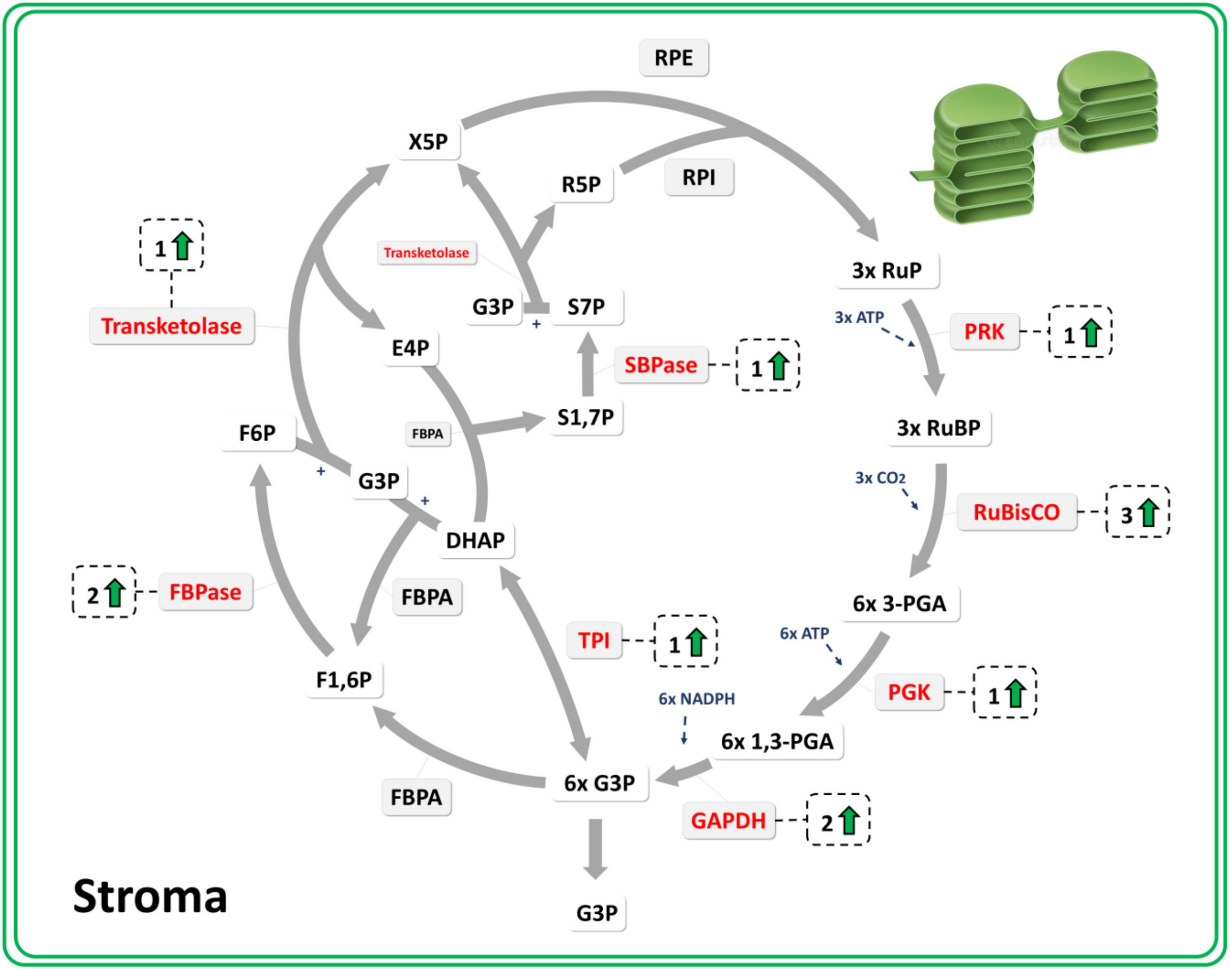
Schematic representation of the Calvin–Benson cycle in the bark of *Eucalyptus grandis* comparing 4- and 12-year-old plants. The diagram illustrates the enzymatic steps involved in the Calvin–Benson cycle. Numbers with upward-pointing arrows indicate the proteoforms of each enzyme that are differential in abundance (fold change > 1.5, *p-*value < 0.05) in bark tissue between 4- and 12-year-old plants. Enzymes displayed in red are those associated with significant functional terms identified in the proteomic analysis.

**Figure 3** shows these metabolic pathways, highlighting enzymes with at least one differentially more abundant proteoform in red. A fold change >1.5 and a Student’s *t*-test comparison were conducted to determine which processes were more active in each tissue and/or developmental stage. In general, 11 proteoforms were more abundant in the 4-year-old xylem, whereas 19 were more abundant in the 12-year-old samples. In the bark, 22 proteoforms were more abundant in 4-year-old trees, compared to only 8 in 12-year-old trees. Additionally, the relative abundance of all proteoforms from carbon metabolism is shown in **Supplementary Figure 1**. While no distinct overall pattern was observed, a slight trend emerged, suggesting that glycolysis and fermentation pathways were more abundant in 12-year-old samples, whereas the TCA cycle was more prominent in 4-year-old trees, as shown in **Figure 4**. This metabolic pathway, crucial for carbon fixation and energy balance, displayed 13 proteoforms that were more abundant in 4-year-old trees than in 12-year-old trees. The sum of the mean abundance of these proteoforms is shown in **Supplementary Figure 2**, reinforcing the view that central carbon metabolism and Calvin–Benson cycle-related reactions are more prominently represented in younger plants at the proteomic level.

## 4 Discussion

### 4.1 Chloroplast abundance in bark and its potential functional role

**Figure 1** not only illustrates a higher number of chloroplasts in the bark of 4-year-old eucalyptus trees but also provides insights into their spatial distribution within the tissue. The observed accumulation of chloroplasts near the outermost region of the bark is likely influenced by light availability, a strategy in regulating the position and functionality of chloroplasts in different plant tissues (Aschan and Pfanz, 2003; Burrows and Connor, 2020). This localization and abundance support the idea that these chloroplasts contribute to corticular photosynthetic capacity, a process that has been shown to supplement carbon assimilation in various tree species (Wittmann et al., 2001; Cernusak and Hutley, 2011). In *Eucalyptus*, the higher chloroplast counts in younger bark, combined with the enrichment of Calvin–Benson cycle enzymes, point to a particularly active chloroplast-associated metabolism in juvenile stems. Additionally, the presence of chloroplasts in the phloem tissues is more evident in younger plants (**Figure 1A1**). This observation suggests a greater physiological demand for corticular photosynthesis in rapidly growing trees, potentially due to an increased need for ATP and carbohydrate production (De Roo et al., 2020). Specially for a fast-growing genus like *Eucalyptus*, younger trees exhibit high metabolic activity and accelerated growth rates, which may surpass the capacity of foliar photosynthesis to supply adequate sugar resources (Dominguez and Niittylä, 2022). Therefore, bark chloroplasts in these juvenile tissues may function as an auxiliary source of photoassimilates to sustain high energy demands.

Another possible explanation for the presence of chloroplasts in the intersection of the cambial region and the phloem tissues is the characteristic hypoxic environment of woody stems. Tree stems often present elevated CO_2_ concentrations and oxygen limitations due to restricted gas exchange and the continuous influx of CO_2_ from root respiration (De Roo et al., 2020; Salomón et al., 2024). Under such conditions, chloroplasts have been proposed to act as local O_2_ producers, supplying oxygen to inner tissues and facilitating mitochondrial respiration through the TCA cycle (Kocurek et al., 2020; Wittmann and Pfanz, 2014). The enrichment of hypoxia-related processes in our dataset, together with the presence of chloroplasts at the interface between phloem and cambial tissues, is consistent with this view of bark chloroplasts contributing to the metabolic environment of woody stems. This mechanism aligns with the presence of chloroplasts in the bark of 12-year-old trees (**Figure 1A2**), suggesting that hypoxia is a consistent factor throughout the plant’s life cycle (León et al., 2020). On the other hand, no evidence of chloroplasts was found in xylem tissue at either age (**Figures 1B1, B2**). This absence is likely due to the inability of light to penetrate deeply into internal tissues (Wittmann et al., 2001), as well as the lower cellular specialization of xylem compared to phloem and vascular cambium. Xylem is primarily adapted for water conduction and structural support rather than metabolic activity, making the presence of functional chloroplasts less relevant in this tissue (Plavcová and Jansen, 2015).

### 4.2 Proteome differences between bark and xylem across developmental stages in *E. grandis*

The comparison between both tissues at two different ages highlighted key metabolic shifts towards different stages of plant development (**Figure 2**). Overall, a consistent enrichment of hypoxia-related processes was observed across all tissues and ages, reinforcing the well-established notion that the vascular system of arboreal plants is characterized by a persistent low-oxygen environment combined with a high metabolic activity associated with vascular function (Van Dongen and Licausi, 2015; León et al., 2020).

A closer analysis into 4-year-old trees (**Figures 2B1, B2**) shows that hypoxia-related terms were among the most enriched in both bark and xylem, suggesting that younger tissues experience a stronger hypoxic stress. This could be linked to the higher respiratory demands of actively expanding cells, leading to increased oxygen consumption and more pronounced hypoxia (Bailey-Serres and Voesenek, 2010). Moreover, while xylem tissue under this age seems to focus on plant development, growth, and signaling process, bark tends to have an even higher response to hypoxia responding to more environmental stimuli such as response to fungus. These findings raise important questions on how plant vascular tissues may contribute to protect the plant against pathogens (Nath et al., 2020; Macháčová et al., 2024).

The changes in protein abundance in the 12-year-old xylem show a metabolic shift towards the younger tissue. This shift indicates a transition from a metabolism focused on tissue expansion to one primarily involved in cellular maintenance and stress adaptation (**Figure 2C1**). The presence of oxidative stress-related pathways suggests that mature xylem presents a higher degree of reactive oxygen species (ROS) accumulation, likely due to reduced metabolic plasticity and prolonged exposure to environmental fluctuations (Mittler et al., 2022). Similarly, the bark also differs from younger to older trees in enriched functions. The bark of 12-year-old trees displayed a strong enrichment of carbohydrate and lignin metabolism-related processes (**Figure 2C2**). This suggests the existence of metabolic reprogramming; the bark transitions from being a site of active growth and hypoxia response to becoming a more storage-oriented and structurally reinforcing tissue. The increased lignin metabolism in older bark could be linked to enhanced secondary cell wall deposition, a process associated with increased mechanical support and defense against pathogens (Malavasi et al., 2016).

These results aligned with the higher density of chloroplasts in the bark of 4-year-old trees, shown in **Figure 1**, corresponding to the stronger enrichment of hypoxia response and growth-related pathways. This pattern is consistent with the idea that corticular photosynthesis contributes to the oxygen balance of developing stem tissues (Wittmann and Pfanz, 2014). Despite the fact that no direct measure was carried out, our proteomic approach shows a greater abundance of proteoforms from the TCA cycle (**Figure 3**) and Calvin–Benson cycle (**Figure 4**) and, together with hypoxia-associated enriched GO terms in young barks, indicates that this coupling between bark chloroplasts and respiratory metabolism is particularly relevant in young *Eucalyptus* stems. In older bark, the reduced presence of chloroplasts coincides with a metabolic shift towards carbohydrate processing and storage, consistent with the view that, as trees mature, bark functions increase as a protective and storage tissue rather than a photosynthetically active one (Burrows and Connor, 2020). These findings highlight how *E. grandis* dynamically optimizes its proteome to meet shifting physiological demands across its lifespan.

### 4.3 Metabolic plasticity in vascular tissues: fermentative pathways supporting energy demands across ages and tissues

**Figure 3** illustrates the interconnected pathways of glycolysis, alcoholic fermentation, and the TCA cycle, highlighting enzymes with at least one differentially abundant proteoform (fold change > 1.5, *p*-value < 0.05) across various tissues and ages. In arboreal species like *E. grandis*, these metabolic pathways are crucial for energy production, growth, and adaptation to environmental stresses. Glycolysis serves as a fundamental anaerobic process, breaking down glucose to provide energy and metabolic intermediates (Chaudhry and Varacallo, 2023). The TCA cycle further oxidizes these intermediates, generating ATP and reducing equivalents essential for various cellular functions (Arnold et al., 2022). Alcoholic fermentation, although less efficient in ATP production, enables energy generation under hypoxic conditions, which can occur in woody tissues (Fredlund et al., 2006; Bernacki et al., 2023).

The analyses showed differences in the proteomic profile in the different tissues. In the case of the xylem of 12-year-old plants, various enzymes showed an increase in abundance, although ADH exhibits three proteoforms more abundant in the 4-year-old xylem, none in the 4-year-old bark, and two in both the xylem and bark of 12-year-old tissues. This pattern suggests that in older tissues, which possess fewer chloroplasts, components of the ethanolic fermentation pathway are more prominently represented and may provide an alternative route for ATP generation to meet the energy demands of vascular tissues under hypoxic conditions. The production of multiple proteoforms from a single gene broadens plant functional plasticity, enhancing responsiveness to fluctuating environments (Kosová et al., 2021). Furthermore, the ability to overcome hypoxia conditions is well established in other organisms (Dashko et al., 2014) and is documented across diverse plant contexts (Geigenberger, 2003; Bailey-Serres and Voesenek, 2010). Here, the absence of differentially abundant ADH proteoforms in the 4-year-old bark could imply that sufficient oxygen supply is present in these tissues, reducing the reliance on fermentative pathways. On the other hand, the increased ADH proteoforms in xylem might indicate that rapid growth necessitates additional energy, potentially surpassing the capacity of chloroplast-rich tissues.

Conversely, ALDH shows two more abundant proteoforms in the 4-year-old bark and two in the 12-year-old xylem. The elevated ALDH proteoforms in the younger bark suggests an active generation of Acetyl-CoA, aligning with the observation of 10 more abundant TCA cycle proteoforms in the 4-year-old bark. ALDH facilitates the conversion of acetaldehyde to acetate, producing NADH and contributing to acetyl-CoA formation, a key TCA cycle substrate (Van Rossum et al., 2016). This pattern is compatible with an enhanced contribution of ALDH-related reactions to Acetyl-CoA supply and mitochondrial metabolism in these tissues, in line with the higher representation of TCA cycle proteoforms in younger bark and the intense energetic demands associated with rapid growth and development (Vera-Vives et al., 2024; Sajib et al., 2023). Similarly, the presence of two ALDH proteoforms in the 12-year-old xylem may indicate an adaptive strategy by the plant, enhancing distinct proteoforms to sustain mitochondrial respiration through the TCA cycle. This aligns with the observation that the relative abundance of ALDH seems not to differ between ages, while TCA cycle proteoforms tend to be more abundant in younger tissues (**Supplementary Figure 1**). Thus, the presence of nine TCA cycle proteoforms in the 12-year-old xylem suggests a metabolic adjustment aimed at maintaining ATP production under hypoxic conditions, emphasizing the plant’s capacity to optimize energy generation in aging tissues and environmental conditions (De Col et al., 2017; Barreto et al., 2022). Furthermore, younger tissues exhibit higher abundance of proteoforms involved in the Calvin–Benson cycle, as shown in **Figure 4** and **Supplementary Figure 2**. Although direct measurements of photosynthetic rates in stem tissues would be required to fully quantify this process, our data indicate that enzymes associated with the carbon fixation, reduction, and regeneration phases of the Calvin–Benson cycle are strongly represented in rapidly growing plants. This aligns with age-dependent differences in photosynthesis-related metabolism reported in other species (Li et al., 2022). The increased photosynthetic capacity in cortical tissues may alleviate hypoxic stress in vascular regions, facilitating efficient mitochondrial respiration and supporting the energetic demands of rapid growth. This is consistent with the presence of more abundant ADH proteoforms in different ages and tissues, indicating that fermentative pathways are strongly represented in the vascular proteome of *E. grandis* and contribute to the metabolic plasticity that supports wood formation in hypoxia-prone environments.

Collectively, our results support the view that *E. grandis* recruits alternative metabolic strategies in vascular tissues to meet the energetic demands associated with rapid early growth, while operating under a hypoxic microenvironment characteristic of developing woody tissues. In this context, the concurrent enrichment of glycolytic and ethanolic fermentation-related proteoforms in inner stem tissues is consistent with a compensatory ATP/NAD^+^ regenerating metabolism when oxygen availability constrains mitochondrial respiration. By combining chloroplast distribution with paired bark and xylem proteome signatures across developmental stages, our study connects corticular photosynthetic capacity with stem oxygen availability and carbon metabolism, providing candidate pathways for hypothesis-driven studies of internal stem O_2_ and CO_2_ dynamics during xylogenesis. Recent advances have begun to quantify whole-stem O_2_ exchange and radial O_2_ profiles under dark and light conditions (Natale et al., 2024), and our dataset contributes complementary molecular signatures that can be prioritized for targeted validation (e.g., isolated stem chloroplast assays, photosynthetic efficiency measurements under controlled irradiance, and paired O_2_/CO_2_ microprofiling). In the longer term, such validated signatures may enable more robust monitoring of stem metabolic states during xylogenesis and guide hypothesis-driven screening across contrasting genotypes, thereby strengthening the translational relevance of stem physiology to tree improvement efforts.

## Data Availability

The proteomics datasets have been deposited in the ProteomeXchange Consortium via the PRIDE repository under accession number PXD067762.
