# Supplementary figures and images for: Comparative proteomics of bark and xylem provides insights into age-dependent corticular photosynthesis in Eucalyptus grandis
Source: Front Plant Sci. 2026 Feb 4;17:1701400. doi: 10.3389/fpls.2026.1701400 (PMC12913457; doi:10.3389/fpls.2026.1701400)

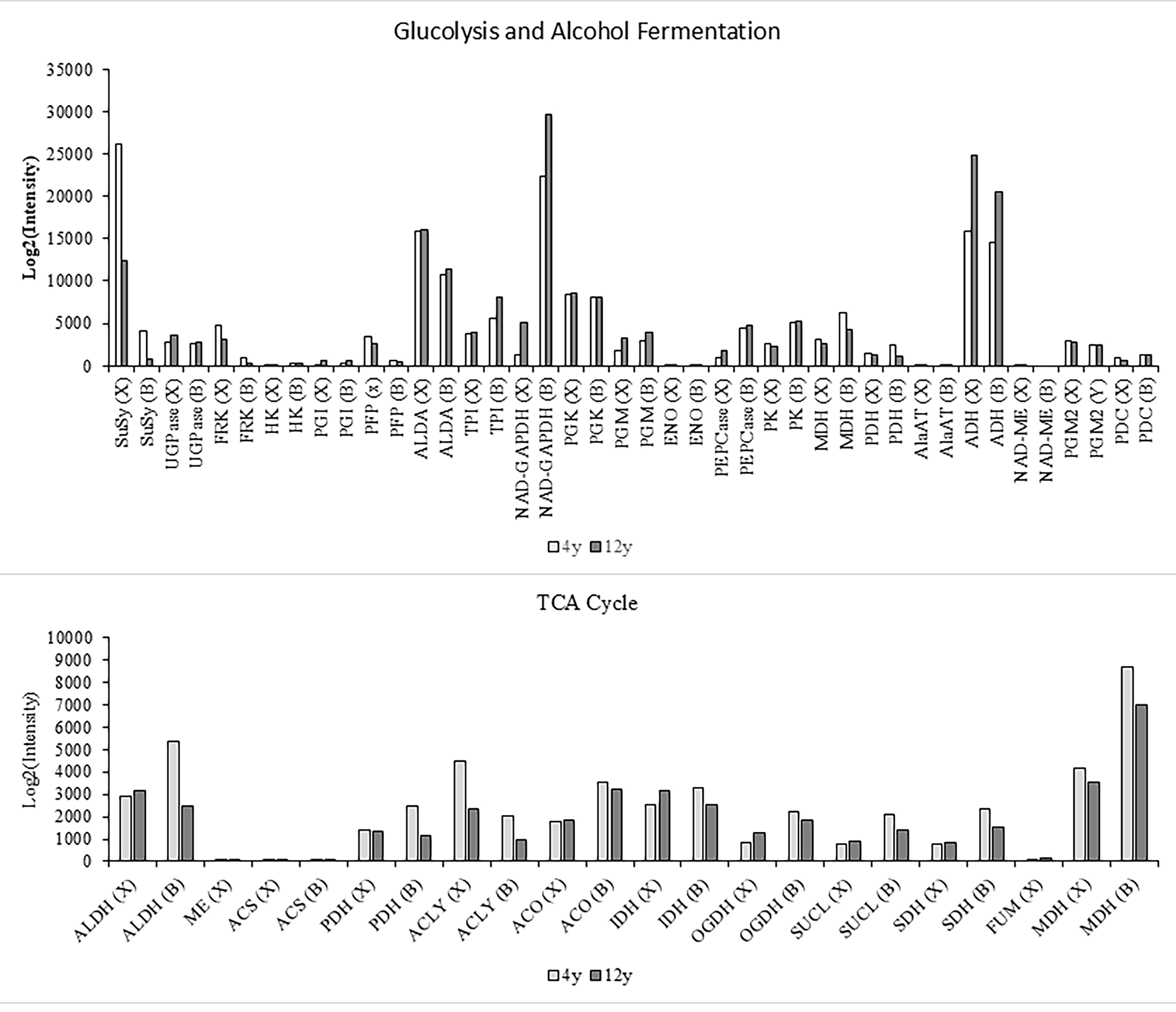

Supplement: Supplementary file 1 [file Image1.png]

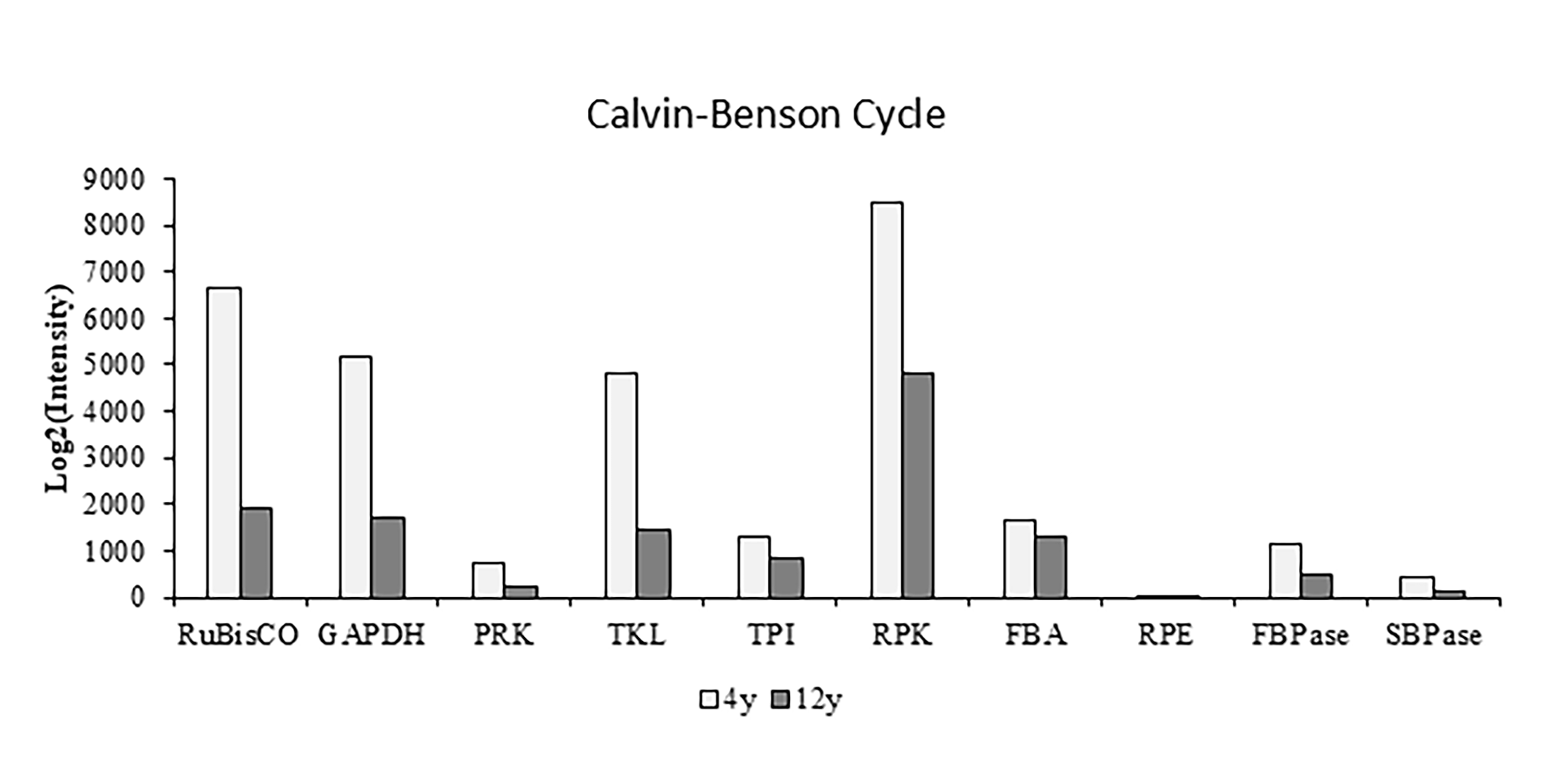

Supplement: Supplementary file 2 [file Image2.png]
